# The effect of type 2 diabetes mellitus and obesity on muscle progenitor cell function

**DOI:** 10.1186/s13287-019-1186-0

**Published:** 2019-03-21

**Authors:** Shuzhi Teng, Ping Huang

**Affiliations:** The Key Laboratory of Pathobiology, Ministry of Education, Norman Bethune College of Medicine, Jilin University, 126 Xinmin Street, Changchun, Jilin 130021 People’s Republic of China

**Keywords:** Satellite cell, muscle progenitor cell, T2DM, Obesity

## Abstract

In addition to its primary function to provide movement and maintain posture, the skeletal muscle plays important roles in energy and glucose metabolism. In healthy humans, skeletal muscle is the major site for postprandial glucose uptake and impairment of this process contributes to the pathogenesis of type 2 diabetes mellitus (T2DM). A key component to the maintenance of skeletal muscle integrity and plasticity is the presence of muscle progenitor cells, including satellite cells, fibroadipogenic progenitors, and some interstitial progenitor cells associated with vessels (myo-endothelial cells, pericytes, and mesoangioblasts). In this review, we aim to discuss the emerging concepts related to these progenitor cells, focusing on the identification and characterization of distinct progenitor cell populations, and the impact of obesity and T2DM on these cells. The recent advances in stem cell therapies by targeting diabetic and obese muscle are also discussed.

## Background

The incidence of overweight has been increasing dramatically worldwide due to increased uptake of high-calorie food, lack of physical activity, and genetic predisposition. Accordingly, obesity and its associated metabolic diseases such as type 2 diabetes mellitus (T2DM) have become an epidemic health threat and economic burden [1]. According to a recent study, by 2025, global obesity prevalence is expected to reach 18% in men and surpass 21% in women [2]. From 1980 to 2014, global age-standardized diabetes prevalence increased from 4.3 to 9.0% in men and from 5.0 to 7.9% in women [3]. Both genetic and environmental factors play pivotal roles in the pathogenesis of T2DM, among them obesity is a major risk factor—around 50% of obese subjects will develop T2DM at some stage [4]. While insulin resistance in peripheral tissues is often an early sign of developing diabetes, pancreatic β cell function is damaged gradually during disease progression. Over time chronic hyperglycemia and hyperlipidemia impair cellular functions, which eventually lead to various complications including diabetic retinopathy, nephropathy, neuropathy, diabetic foot, and cardiovascular diseases [4].

A major metabolic defect associated with T2DM is the failure of proper glucose utilization by peripheral tissues such as skeletal muscle and adipose tissue, the primary targets of insulin-stimulated glucose uptake. In healthy humans, 70–80% of glucose uptake occurs in skeletal muscle in the postprandial state through both insulin-dependent phosphoinositide-3-kinase-protein kinase B (PI3K-PKB/AKT) pathway and insulin-independent glucose-stimulated Baf60c-Deptor-AKT pathway [5, 6]. Moreover, studies of genetically predisposed individuals indicate that insulin resistance in muscle is the primary or initiating defect leading to the ultimate development of T2DM [7, 8]. Skeletal muscle not only plays an important role in the pathogenesis of T2DM but also undergoes significant structural, metabolic, and functional changes under obese and diabetic conditions, such as muscle atrophy [9, 10], fiber-type transition [11], impaired glucose uptake [12], glycogen synthesis [13, 14], fatty acid oxidation [15], and altered myokine secretion [16, 17], which ultimately lead to muscle weakness and poor exercise performance.

Many of the morphological features of muscle atrophy resemble those seen in sarcopenia, an age-associated loss of skeletal muscle mass and function [18]. Both muscle atrophy and sarcopenia are characterized by a decrease in myofiber size and muscle mass, and the ensuing loss of muscle strength. Loss of appendicular lean mass and reduced skeletal muscle strength are commonly observed in T2DM patients despite gender and ethnicity and the incidence increases with aging [19, 20]. It is estimated that sarcopenia is present in about 5 to 10 % of persons over 65 years of age [21], whereas the T2DM patients have two to three times higher prevalence of sarcopenia than non-diabetic individuals [22, 23] due to glucose toxicity, insulin resistance, and oxidative stress [21]. In addition, intermuscular adipose tissue infiltration is increased in persons with obesity, diabetes, and peripheral neuropathy [24]. This ectopic fat accumulation is associated with impaired muscle function and forms the basis of obese sarcopenia [24].

A key component to the maintenance of skeletal muscle integrity and plasticity is the presence of muscle progenitor cells, including satellite cells, fibroadipogenic progenitors, myo-endothelial cells, and other interstitial progenitor cells [25–29]. These heterogenous groups of cells are endowed with multilineage developmental potential and may contribute to the pathogenesis as well as the treatment of muscle diseases.

Accumulating evidence indicates that diabetic and obese conditions not only cause dramatic structural, metabolic and functional changes of skeletal muscle fibers but also display detrimental effects on these progenitor cells [30, 31]. Therefore, fully characterization of distinct progenitor cell populations and understanding the impact of aging, obesity, and T2DM on these cells will aid to extend our understanding of these health conditions and shed light on developing novel therapeutic interventions.

## Effect of T2DM and obesity on skeletal muscle stem cell—satellite cell

### Satellite cell discovery and function

The satellite cell was first identified by transmission electron microscopy on frog muscle and named after its anatomical localization: mononuclear cell wedged between basal lamina and the plasma membrane of myofiber, like a “satellite” cell “orbiting” the muscle fiber when viewed in cross-section [25]. Shortly afterwards, the satellite cell was found in other vertebrates including human [32]. Subsequent studies proved that satellite cells are capable of proliferation and myogenic differentiation in vitro and in vivo [33, 34]. Recent studies further demonstrated the self-renewal capacity of satellite cells after transplantation [35, 36]. All these data qualify satellite cells as skeletal muscle-specific stem cells.

Identification of the paired-box transcriptional factor Pax7 being specifically expressed in satellite cells enables researchers to use Pax7 lineage tracing to study the satellite cell function [37]. Accumulated data from Pax7 expressing cells indicate that the satellite cell population serves as a major contributor to the postnatal muscle growth and repair after injury or disease [38, 39]. In adulthood, satellite cells remain quiescent under normal conditions. Upon injury or in diseased states, satellite cells are reactivated, proliferating to generate a pool of myoblasts, which then differentiate and fuse with damaged fibers or fuse with each other to generate entirely new myofibers. Meanwhile, some myoblasts remain undifferentiated and return to the quiescent state to replenish the satellite cell pool. During muscle development and regeneration, myogenic regulatory factors (MRFs) Myf5, MyoD, MRF4, and myogenin are activated for entry of satellite cells into the myogenic program [40]. The importance of satellite cells for muscle regeneration is reinforced by genetic ablation of Pax7 expressing cells after acute injury. Lepper and colleagues found that such elimination completely blocked muscle regeneration, thus verified that satellite cells, as adult stem cells, are indispensable for acute injury-induced muscle regeneration [39].

### The proliferation and differentiation of satellite cells are attenuated in diabetic muscles

The impairment of muscle regeneration was observed in animal studies under hyperglycemia and/or lipotoxicity conditions, but the detailed changes especially alterations of satellite cells vary from study to study, and some results are even conflicting (Table 1). Using a mouse model of insulin resistance achieved by high-fat diet (HFD) feeding for 8 months, Hu et al. observed smaller regenerating myofibers plus more collagen deposition after cardiotoxin injury [30]. However, satellite cell activation or proliferation was intact as assessed by bromodeoxyuridine (BrdU; an analog of the nucleoside thymidine) incorporation and by the expression of myogenic transcription factors. Instead, the deficits in muscle regeneration were principally related to increased expression of phosphatase and tensin homolog (PTEN), which reduced phosphatidylinositol (3,4,5)-trisphosphate (PIP3) in muscle, inhibited AKT signaling, and impaired myofiber maturation [30].

**Table 1:** Effect of insulin resistance on skeletal muscle development and regeneration in animal models

Animal	Treatment	Start age	Chow (gm%)	Muscle mass/size	Muscle regeneration	SC content	SC proliferation	Mechanism	Reference
C57BL6 mice	HFD for 8 months	6-week-old	23% protein, 35.8% fat, and 35.5% carbohydrate	N/A	Smaller regenerating myofibers plus more collagen deposition	N/A	No change	Increased expression of PTEN, which lowers muscle PIP3	[30]
C57BL6 mice	HFD for 3 weeks	3-week-old	26.2% protein, 34.9% fat, and 26.3% carbohydrate	Decreased muscle mass	Reduced regenerative capability	Decreased	N/A	N/A	[41]
C57BL6 mice	HFD for 12 weeks	4-week-old	60% kcal from fat	N/A	Normal	N/A	No change	N/A	[55]
C57BL6 mice	HFD for 6 weeks	4-week-old	18.3% protein, 60.9% fat, and 20.1% carbohydrate (kcal%)	No change	Delayed	No change	Reduced	SCs do not respond to HGF activation	[42]
Ob/ob and db/db mice	Normal chow		6% kcal from fat	N/A	Delayed	N/A	Reduced	N/A	[55]
OZR	Normal chow			Smaller skeletal muscle size	N/A	SC% no change	Decreased	Reduced Akt signaling	[45]
OZR	Normal chow			N/A	N/A	N/A	Normal	N/A	[46]

*SC* satellite cell, *HFD* high-fat diet, *HGF* hepatocyte growth factor, *OZR* obese Zucker rats, *N/A* not available

On the other hand, HFD-feeding 3-week-old mice for just 3 weeks resulted in overweight, decreased satellite cell content and muscle mass, and reduced regenerative capability [41]. In another study, HFD-feeding 4-week-old mice for 6 weeks led to delayed myofiber regeneration due to attenuated satellite cell proliferation even though satellite cell content remained unchanged [42]. In agreement with these reports, Fu et al. showed that C57BL/6 mice fed with a 60% HFD for 3 months became obese and muscle injury induced by cardiotoxin resulted in impeded satellite cell activation and proliferation, and fewer regenerated fiber formation in obese mice [43]. Further analysis revealed that decreased 5′ AMP-activated protein kinase (AMPK) α1 activity in satellite cells accounted for the impaired muscle regeneration [43].

The Obese Zucker rat (OZR), a model of metabolic syndrome resulted from a homozygous missense mutation of the leptin receptor gene [44], displays smaller skeletal muscle size than the Lean Zucker rat (LZR) [45]. This defect has been attributed to a significant decrease in satellite cell proliferative capacity though the proportion of quiescent satellite cells remained unchanged. However, compensatory loading on OZR muscle can restore satellite cell proliferation, Akt signaling, MyoD, and myogenin expression [45]. In contrast, Scarda et al. demonstrated that satellite cells isolated from OZR did not show any difference in terms of proliferation rate and differentiation potential compared to their lean littermates [46]. Taken into consideration that increased protein degradation has also been shown to contribute to muscle atrophy in OZR [47], future studies are necessary to delineate the precise underlying mechanisms. Of note, one major machinery in this setting is the ubiquitin-proteosome system. The two major ubiquitin ligases Atrogin1 (also known as MAFbx or FBXO32) and muscle ring-finger protein-1 (MuRF1) are both upregulated in diabetic and obese-induced atrophy muscle [10]. Atrogin1 targets MyoD and eukaryotic translation initiation factor 3 subunit F (eIF3-f) for protein degradation [48, 49], whereas MuRF1 induces degradation of a group of proteins important for maintaining sarcomere integrity such as actin, telethonin, myosin light, and heavy chains [50–52]. More detailed cellular and molecular mechanisms of skeletal muscle atrophy and sarcopenia have been exquisitely reviewed elsewhere [53].

Ob/ob and db/db mice have mutations in the genes encoding leptin and the leptin receptor, respectively. They are obese and diabetic and are well-characterized models for type 2 diabetes [54]. Following cardiotoxin injury, both ob/ob and db/db mice showed impaired muscle cell proliferation, decreased myoblast accumulation, and delayed muscle regeneration [55]. In comparison, such changes were not seen in 3-month-old HFD-fed diabetic and obese mouse, which is a less severe model of insulin resistance [55]. The above-mentioned disparities are possibly due to genetic model difference, variations in HFD composition and diet length, and type of analysis performed (Table 1). Moreover, severity of insulin resistance, inflammatory response, fiber-type transition, glucose and fatty acid metabolic changes etc., though unelucidated, could have an impact on the regenerative process and satellite cell functionality. Thus, further investigations are needed to clarify this issue.

In addition to the reduced myogenic potential, satellite cells isolated from T2DM patients maintained other diabetic phenotypes during in vitro culture, such as impaired glucose uptake, decreased glycogen synthesis, reduced fatty acid oxidation, and increased inflammatory response and insulin resistance [12, 56, 57]. These results indicate that the insulin-resistant phenotype is intrinsic to muscle satellite cells and justify the use of satellite cell culture as a tool to study regulatory mechanisms in obesity and T2DM in humans ex vivo.

Skeletal muscle is gaining recognition as an endocrine organ capable of synthesis and secretion of myokines. Human skeletal muscle satellite cells obtained from T2DM subjects were differentiated into myotubes, which secreted elevated amount of myokines including IL-6, IL-8, IL-15, TNFα, follistatin, and monocyte chemotactic protein (MCP)-1 compared to control myotubes [17]. These secreted factors may have impact on multiple tissues and contribute to the development of diabetic phenotypes. A complete analysis of secretomes between T2DM and normal myotubes may identify more aberrantly secreted myokines as shown by proteomic studies performed in palmitate-induced insulin-resistant muscle cell lines [58, 59].

### Transdifferentiation of satellite cells in obese and diabetic conditions

Satellite cells have been reported to be multipotent and can differentiate into myocytes, adipocytes, and osteocytes in vitro [60–62]. Adipogenic differentiation can be induced by inhibition of Wnt signaling [63], high oxygen pressure [64], and growth in adipogenic media [61] and can be enhanced in aged muscle [65]. In line with these findings, satellite cells of obese animals displayed an enhanced adipogenesis under adipogenic conditions that may result from Wnt10b downregulation [46]. Similarly, high-glucose exposure in vitro induced adipogenic differentiation of muscle-derived stem cells including satellite cells [66].

More recently, satellite cell transdifferentiation capacity to adipocyte was analyzed using the *Cre-loxP* system for lineage tracing with the *cre* gene driven by the MyoD promoter. MyoD^Cre^-labeled cells (EYFP^pos^) derived from MyoD^Cre^:R26R^EYFP^ skeletal muscle represent 98% of Pax7^pos^ satellite cells [67]. However, these EYFP^pos^ cells do not spontaneously adopt an adipogenic fate. Under adipogenesis-inducing conditions, EYFP^pos^ satellite cells accumulated cytoplasmic lipid but maintained myogenic protein expression and did not undergo complete adipogenic differentiation [68], suggesting that these adipocyte-like cells are not intrinsic adipocytes. Consistent with this finding, satellite cell transition to adipocyte-like cell was also demonstrated when Lkb1 gene was specifically knocked out in MyoD progenitors, in which myogenic gene expression was not downregulated [69]. Nevertheless, Lkb1-null myofibers accumulated excessive lipids in vivo in response to HFD feeding, suggesting that alterations in lipid metabolism in satellite cells could lead to physiological consequences in the adult muscle [69]. In this regard, excessive lipid accumulation in satellite cells derived from obese animals or cultured under high glucose conditions probably reflects a metabolic adaptation rather than a cell identity switch to adipocytes. Taken together, the in vivo lineage analysis of satellite cell fate under obese/diabetic conditions will further clarify its adipogenic potential.

Accumulating evidence reveals that high intramyocellular lipid (IMCL) content is associated with insulin resistance in aging [70], T2DM [71], and obesity [72]. Paradoxically, elite endurance athletes, who possess high insulin sensitivity, have similar IMCL levels as insulin-resistant obese or T2DM subjects [73]. It is not yet clear which underlying mechanism explains this paradox, but the lipid composition, metabolites and associated proteins may affect the relationship between IMCL and insulin sensitivity [74]. Nevertheless, moderate exercise training in obese older adults can improve insulin sensitivity and enhance muscle oxidative capacity in conjunction with favorable lipid repartitioning [75].

## Effect of T2DM and obesity on skeletal muscle interstitial progenitor cells

Muscle-resident interstitial progenitor cells often possess multipotent differentiation ability, and many of them (that we will discuss further below) have myogenic differentiation ability that endowing them with therapeutic potential. It is of note that fibroadipogenic progenitors are not myogenic, and they account for the fatty degeneration of the diabetic/obese muscle. Nevertheless, these interstitial progenitors can coordinate with each other or with satellite cells to play important roles in muscle repair. Understanding how T2DM and obesity affect these progenitor cells will help developing strategies against muscle wasting and dysfunction in this setting.

### Fibroadipogenic progenitors (FAPs)

**Fig. 1:**
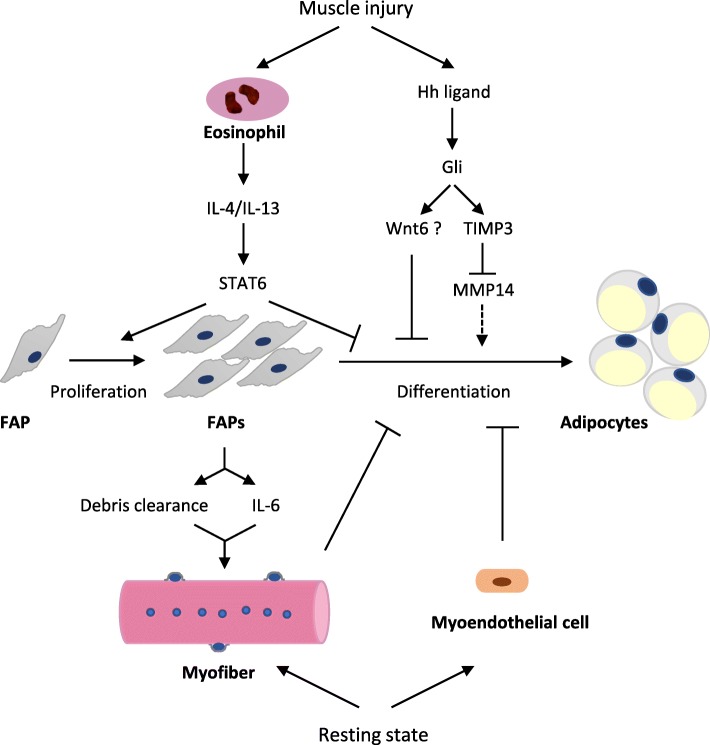
Illustration of the mechanisms for the adipogenic fate determination of FAP in skeletal muscle. Eosinophils infiltrate early during muscle injury, secrete IL-4/IL-13, and subsequently stimulate STAT6 to promote FAP proliferation, while inhibiting its adipogenic differentiation. Activation of Hh signaling also prevents the conversion of FAP to adipocyte. Meanwhile, the direct interaction of FAP with intact myofiber or myo-endothelial cell can prevent its differentiation into adipocyte at resting state. Upon muscle damage, FAPs proliferate dramatically to help debris clearance and induce myogenic cell differentiation. FAP, fibroadipogenic progenitor; STAT6, signal transducer and activator of transcription 6; Hh, hedgehog; TIMP3, tissue inhibitor of metalloproteinases 3; MMP14, matrix metallopeptidase 14

Excessive intermuscular fat deposition is observed in obese, diabetic, dystrophic, and aging animals and patients and associates with insulin resistance, loss of muscle strength, and other impaired physical functions [31, 76]. To clarify the origin of cell populations account for the fatty degeneration of skeletal muscle, two groups employed fluorescence activated cell sorting (FACS) and identified very similar platelet-derived growth factor receptor α (PDGFRα)^pos^ interstitial cell population, termed fibroadipogenic progenitors (FAPs) [26, 27]. These cells have the potential to produce both fibroblasts and adipocytes but failed to differentiate into myogenic cells [77, 78]. The fate of FAPs in vivo is largely dependent on the environment. Under resting and normal regenerating conditions, FAPs are quiescent and proliferating yet remain undifferentiated, respectively. In both cases, interaction with intact myofiber or adequate proliferating myoblasts or myo-endothelial cells prevents FAPs differentiation into adipocytes (Fig. 1) [26, 79]. Conversely, FAPs gave rise to ectopic white fat when delivered subcutaneously or intramuscularly in a model of fatty infiltration such as glycerol injection [26, 27], or under Duchenne muscular dystrophy (DMD) conditions with severe satellite cell damage [80], or in type 2 diabetic KKAy and obese mice after cardiotoxin-induced injury [76]. Profibrotic cytokines TGFβ and PDGF could stimulate the proliferation and differentiation of FAPs to fibrogenic cells in vitro, and transplanted FAPs solely give rise to collagen type I-producing cells (fibroblasts) in the gamma radiation-induced muscle fibrosis model [77].

The mechanisms for the adipogenic fate determination of FAP are not fully elucidated and may involve multiple signaling pathways. Upon muscle injury, eosinophils are recruited to the injured site, secreting IL-4 and IL-13. These inflammatory signals act through IL-4Rα to stimulate signal transducer of transcription 6 (STAT6), which promotes FAP proliferation, whereas inhibits its differentiation into adipocytes [81]. Hedgehog (Hh) signaling has also been shown to inhibit adipogenesis of both pre-adipocytes and FAPs [82, 83]. The newly identified downstream target in this setting is tissue inhibitor of metalloproteinases 3 (TIMP3), a secreted metalloproteinase inhibitor, that specifically inhibits matrix metallopeptidase 14 (MMP14) to prevent adipogenesis of FAP [83] (Fig. 1).

FAPs also exhibit osteogenic potential upon stimulation with bone morphogenetic protein (BMP) ligands [26] and have recently been shown to be a major cell origin of heterotopic ossification (HO; extraskeletal bone formation) in fibrodysplasia ossificans progressiva (FOP) mouse muscles [84, 85]. Activin A receptor type I (ACVR1) is a BMP type I receptor, and a missense mutation in the glycine-serine activation domain of ACVR1 (R206H) is the underlying cause of FOP [86]. After global postnatal induction of mutated ACVR1 in ACVR1^R206H/+^ transgenic [87] and knock-in mice [88, 89], HO is formed in the skeletal muscle of activin A administration sites [87, 88], or cardiotoxin injury sites [89], or even spontaneously in mirroring sites that commonly ossify in FOP patients [88, 89]. The non-genetic forms of HO are particularly common among patients with traumatic injuries, burn injury, and soft tissue damage [90]. The incidence of HO was reported to be over 60% in a cohort of combat-injured patients [90]. Whether FAPs contribute to HO in this setting remains to be elucidated, nevertheless, FAP may represent a potential target for cell-based therapy for FOP.

FAPs play pivotal roles in maintaining muscle homeostasis and promoting muscle regeneration in vivo. Upon muscle damage, FAPs proliferate dramatically in the first three days, exhibit around tenfold increase of IL-6 expression, and induce myogenic cell differentiation [27]. A study by Mozzetta et al. further explored FAP regulation of myogenesis in young and old mdx mice. Ex vivo co-culture and in vivo co-transplantation of FAPs and satellite cells showed that FAPs derived from young but not old mdx muscle enhanced adjacent satellite cell differentiation. In addition, the progressive loss of satellite cell regenerative capacity was partially attributed to paracrine reduction of follistatin by FAP in old mdx muscle [91]. Of note, paracrine action of IL-6 and follistatin has been implicated in myoblast differentiation [92, 93]. The functions performed by FAPs are not restricted to supporting myogenesis. Heredia et al. affirmed that muscle damage stimulated FAP proliferation in vivo via IL-4 signaling, and FAPs were capable of phagocytizing necrotic cellular debris in regenerating muscle [81]. Finally, muscle-specific ablation of adipocyte protein 2 (ap2, a.k.a. fatty acid binding protein 4; FABP4) lineage cells, which predominantly give rise to FAPs, leads to dramatic inhibition of myogenic differentiation, reduction of regenerating myofiber number and size in cardiotoxin-injured muscle [94]. Similarly, conditional ablation of Tcf4 lineage fibroblasts, which largely overlap with FAPs in skeletal muscle, results in impaired muscle regeneration with premature satellite cell differentiation, depletion of the satellite cell pool and smaller regenerating myofibers [95]. Collectively, these data further support the notion that FAPs are required for muscle regeneration.

Human putative FAPs were identified in muscle interstitium and could be isolated using several cell surface markers including PDGFRα, which identifies both mouse and human FAPs (Table 2) [96–98]. Human FAPs possess fibrogenic, adipogenic, and osteogenic capacities and in DMD patients FAPs contribute to both pre-adipocytes expressing PPARγ and fibroblasts expressing collagen I. Moreover, the number of PDGFRα^pos^ cells positively correlates with the severity of fibrosis, demonstrating the pathophysiological importance of this cell population [96].

**Table 2:** Major skeletal muscle-resident cell populations

Muscle-resident cell population	Species	Surface marker in majority of cells	Surface marker in subset of cells	In vitro lineage potential	In vivo lineage potential	Reference
Satellite cell	Mouse	Vcam-1 (CD106), Synd3/4, M-Cadherin, SM/C-2.6, Caveolin-1, Calcitonin receptor, β1-integrin, CXCR4, α7-integrin, CD34	c-Met, Jagged-1, CD56	Myogenic, adipogenic and osteogenic	Myogenic	[27, 40, 148–154]
	Human	CD56 (NCAM), M-cadherin, α7-integrin, CD82, CD318	CD146 (MCAM)	Myogenic, osteogenic	Myogenic	[98, 155–158]
FAP	Mouse	PDGFRα, Sca-1, CD34	Tie2	Fibrogenic, adipogenic and osteogenic	Fibrogenic, adipogenic and osteogenic	[26, 27, 77, 85]
	Human	PDGFRα, CD15, CD201	CD166	Fibrogenic, adipogenic and osteogenic	Fibrogenic, adipogenic and osteogenic	[96–98, 159]
Endothelial and myo-endothelial cell	Mouse	CD31, Sca-1, Tie2 (90%), VE-cadherin (90%)	β4-integrin (10%)	Myogenic and adipogenic	Myogenic	[79]
Myogenic-endothelial progenitor	Mouse	CD34, Sca-1		Myogenic, endothelial and adipogenic	Myogenic and endothelial cell	[104]
	Human	CD34, CD56, CD144 (VE-cadherin)		Myogenic, adipogenic, osteogenic, chondrogenic and endothelial cell	Myogenic, osteogenic, chondrogenic and endothelial cell	[28, 105]
Pericyte	Mouse	PDGFRβ, CD146, NG2	Sca-1, CD34, PDGFRα, Nestin	Myogenic adipogenic	Myogenic, adipogenic and pericyte	[108, 110, 111]
	Human	PDGFRβ, CD146, NG2		Myogenic, adipogenic, osteogenic and chondrogenic	Myogenic osteogenic	[109, 112]
PIC	Mouse	Sca-1, CD34	PDGFRα, PDGFRβ	Myogenic, smooth muscle cell and adipogenic	Myogenic	[129, 130, 134]
Twist2+ progenitor	Mouse	β1-integrin, Sca-1, PDGFRα, PDGFRβ		Myogenic and osteogenic	Myogenic	[132]
Interstitial myogenic cell	Mouse	β4-integrin (CD104), β1-integrin (60%), Sca-1 (55%)	CD31, CD34, α7-integrin (34%)	Myogenic	Myogenic	[160]

*VCAM-1* vascular cell adhesion molecule-1, *MCAM* melanoma cell adhesion molecule, *NCAM* neural cell adhesion molecule, *FAP* fibroadipogenic progenitor, *PIC* PW1-expressing interstitial cell, *NG2* neuron-glial antigen

The origin of ectopic adipocytes in diabetic and obese muscle has been investigated recently. Studies from T2DM mice KKAy, db/db, and HFD-fed obese mice showed that ectopic adipocytes accumulated in lower limb were derived from PDGFRα^pos^ progenitors [76]. In agreement with this, adipogenic progenitors from obese human skeletal muscle can be isolated from CD56^neg^CD15^pos^ cellular fraction [99], which is equivalent to PDGFRα^pos^ FAPs [97]. Mature adipocytes derived from muscle FAPs exhibited the phenotype, molecular characteristics, and metabolic properties of white adipocytes. However, these FAP-derived adipocytes were insensitive to insulin signaling, as indicated by lack of phosphorylation of insulin receptor, its downstream targets insulin receptor substrate-1 (IRS-1), AKT, and mitogen-activated protein kinase (MAPK) and inhibition of stimulated glucose uptake [97]. Furthermore, human primary myotubes cultured with conditioned media from CD56^neg^CD15^pos^ adipocytes of obese muscle displayed significant reduction of insulin-stimulated glucose uptake, glycogen synthesis, and glucose oxidation, indicating paracrine factors secreted from FAP-derived adipocytes negatively regulate insulin action in skeletal muscle cells [99]. Future studies should focus on identifying these bioactive molecules.

Since the characterization of FAPs in skeletal muscle several years ago, FAPs gradually emerged as novel targets for treating muscle disorders. For example, imatinib, a tyrosine kinase receptor (including PDGFRα) inhibitor, has been shown to inhibit proliferation and fibrotic differentiation of FAPs via blocking PDGF signaling, resulting in amelioration of the muscular pathology in severe muscular dystrophy mice [100]. Moreover, Cordani and colleagues revealed nitric oxide prevented adipogenic differentiation of FAPs in dystrophic muscle via elevation of miR-27b expression and downregulation of PPARγ expression [80]. In a mouse muscle tendon injury model, TGFβ inhibitor was shown to prevent muscle fatty infiltration and fibrosis by promoting FAP apoptosis [101]. Endurance exercise training induces type II fast to type I slow myofiber conversion. Zeve et al. reported that endurance exercise suppressed adipogenic progenitor proliferation and differentiation into mature adipocytes in vitro and suppressed adipogenesis in vivo in HFD-feeding mice, partially through secretion of R-spondin 3 from slow myofiber, which may activate Wnt signaling to suppress adipogenesis [102]. Muscle fibrosis is also seen in chronic kidney disease (CKD). Using a mouse model for CKD, Dong et al. demonstrated that FAPs account for muscle fibrosis [103], and elevated myostatin production in CKD muscle directly stimulates the proliferation and differentiation of FAPs into fibroblasts via Smad3 signaling, while myostatin inhibition suppresses muscle fibrosis and increases muscle mass [103]. Collectively, these data open up the opportunities of developing novel treatment strategies targeting mesenchymal FAPs to target disorders associated with muscular system. Reciprocally, myofibers can regulate adipose progenitor activity through secreted factors, which could lead to the development of novel therapeutic approaches for obesity and metabolic syndrome.

### Myo-endothelial cells

Besides FAP, other muscle interstitial progenitor cells that can differentiate into adipocytes have been identified. This is a heterogeneous group of cells that can be isolated based on distinct cell surface markers (Table 2) and usually exhibits multipotency including myogenic potential. They are distinct from satellite cells with regard to cellular localization and being largely negative for Pax7 expression when freshly isolated. Most of these cells are associated with muscle vessels. One such kind of cells is called myogenic-endothelial progenitor cells. Tamaki and colleagues showed that FACS-sorted CD34^pos^ Sca-1^pos^CD45^neg^ cells can differentiate into myogenic, endothelial, and adipogenic cells in vitro, whereas yield only myoblasts and endothelial cells when implanted in muscle [104]. Recently, we studied Myf5^pos^CD31^pos^Sca-1^pos^CD45^neg^ myo-endothelial progenitors and revealed that bone morphogenetic protein receptor 1a (Bmpr1a) signaling was essential for myogenic fate determination of these cells, whereas ablation of Bmpr1a increased their adipogenic differentiation potential [79]. Furthermore, the major function of these myo-endothelial cells in vivo is to inhibit intramuscular adipogenesis through cell-autonomous and cell-cell interaction mechanisms (i.e., through inhibition of FAP adipogenic differentiation) [79]. In 2007, a human counterpart of myo-endothelial cells was prospectively isolated using both satellite cell and endothelial cell markers (CD56, CD34, CD144, Table 2) and these cells displayed myogenic, osteogenic, chondrogenic, adipogenic, and angiogenic differentiation potential [28, 105]. More importantly, these myo-endothelial cells regenerated myofibers more efficiently than CD56^pos^ satellite cells in cardiotoxin-injured muscle of severe combined immune-deficient mice [28].

Angiogenesis and myogenesis is tightly coordinated for muscle regeneration and satellite cell survival. Endothelial cells (ECs) strongly promote myogenic cell growth and differentiation and inversely, myogenic cells stimulate EC capillarization and lumenization, indicating the reciprocal stimulation of the two cell types. Endothelial dysfunction is a common finding in diabetic patients and animal models [106], but how the subset of ECs—myo-endothelial cells are affected merits further investigation.

### Pericytes and mesoangioblasts

Pericytes, the contractile mural cells surrounding the endothelial cells of capillaries and microvessels throughout the body, are important for blood flow regulation, microvasculature integrity, and angiogenesis [107]. Pericytes are characterized by the expression of PDGFRβ, CD146 (M-CAM), neuron-glial antigen 2 (NG2), and α-smooth muscle actin. In mouse skeletal muscle, two types of pericytes have been identified. Both of them express above-mentioned pericyte markers, but only type 1 pericytes express the FAP marker PDGFRα and differentiate into adipocytes in vitro and in vivo, whereas type 2 pericytes are myogenic [108]. Human pericytes were also prospectively isolated as CD146^pos^CD34^neg^CD45^neg^CD56^neg^ cells from various tissues and displayed multilineage developmental potential including myogenic differentiation in spite of their origins (Table 2) [109]. Mouse lineage-tracing experiments and postnatal ablation of muscle pericytes demonstrated that muscle pericytes not only commit to reparative angiogenesis, but also contribute directly to postnatal myofiber growth and regeneration [110, 111]. Meanwhile, pericytes tightly regulate satellite cell growth/differentiation and quiescence through secretion of IGF1 and angiopoietin 1, respectively [111]. Intriguingly, human pericyte transplantation into immune-deficient X-linked muscular dystrophy mice through femoral artery-generated numerous functional myofibers expressing human dystrophin and replenished the satellite cell pool, indicating that pericytes can penetrate basal lamina to fuse into myofibers [112]. In summary, human pericytes can be expanded in vitro, genetically modified and delivered systemically, which makes this cell population a great candidate for cell-based therapy to treat muscle diseases.

Pericyte deterioration and apoptosis are found in most of the diabetic microvascular complications such as diabetic retinopathy, nephropathy, neuropathy, and type 2 diabetic muscles [113]. Such changes are often followed by reduced capillary density, which could ultimately block insulin and nutrients to reach myocytes [114] and impaired angiogenesis upon the induction of limb ischemia [115]. Oxidative stress at least in part accounts for the underlying mechanisms by which T2DM impairs pericyte function [115, 116]. As shown in an in vitro experiment, hyperinsulinemia-induced pericyte oxidative stress through upregulation of the NADPH oxidase gene Nox2 and reduction of pericyte tube formation capacity; whereas addition of antioxidant NAC prevented Nox2 upregulation and reversed the phenotype [115]. In another study, skeletal muscle pericytes derived from T2DM patients with critical limb ischemia exhibited deficits in terms of decreased proliferation and reduced myogenic ability and antiangiogenic activity, which were associated with downregulation of the antioxidant enzymes superoxide dismutase 1 and catalase, and activation of the pro-oxidant PKCβII-p66^Shc^ pathway [116]. In addition, muscular pericytes in diabetic patients are prone to adipogenesis at the expense of myogenesis and angiogenesis [116]. Therefore, restoring pericyte angiomyogenic activity holds therapeutic potential in diabetes.

Mesoangioblasts (MABs), first isolated from the mouse embryonic dorsal aorta, have been identified from postnatal skeletal muscle vessels of different species [29]. MABs express endothelial and/or pericyte markers, possess multipotent mesoderm differentiation ability, and can be expanded in vitro. Most importantly, the transplanted MABs home to and regenerate in the injured/dystrophic muscles upon intra-arterial delivery. Both the systemically and locally delivered MABs regenerate muscle fibers [117–119]. These characteristics are quite similar to pericytes, although the relationship between MABs and pericytes is still not fully understood. Skeletal muscle MABs can be isolated from explant cultures of muscle tissue and by selection of a small, round, refractile cell population [29], whereas pericytes are usually isolated by FACS using specific cell surface markers (Table 2) [109]. Even though typical pericyte markers such as NG2 and PDGFRβ were detected in cultured MABs and remained stable over passages, it was not clear whether these MABs expressed pericyte markers immediately after isolation or gained pericyte gene expression profile over time in culture [120]. Nevertheless, in various animal models, donor or self genetically corrected MABs could restore dystrophin expression in 10–70% of myofibers of dystrophic muscle and significantly improved muscle contraction force and motility [117, 121]. These promising studies have led to the first phase I–IIa clinical trial in five DMD patients via intra-arterial transplantation of HLA-matched allogeneic MABs. Cossu et al. demonstrated that this procedure was relatively safe and donor DNA was detected in recipient patients, but no functional improvements were observed [122]. Thus, this study provides a starting point for refining the treatment regiments of MAB therapy.

Mesoangioblasts isolated from muscle biopsies of inclusion-body myositis and facioscapulohumeral muscular dystrophy patients display remarkable myogenic differentiation defect [123, 124]. Whether hyperglycemia and hyperlipidemia are associated with any dysfunction of MABs has not been examined yet. It is well documented that plasma adiponectin content is decreased in obese and/or diabetic individuals [125, 126]. Moreover, adiponectin produced by skeletal muscle is greatly reduced in db/db T2DM mice and in myotubes cultured under hyperglycemic condition [127]. Adiponectin exhibits advantageous effects on MABs including induction of MAB proliferation, migration, and myogenic differentiation and protecting MABs from apoptosis in vitro and in vivo [128]. Therefore, it is feasible to postulate that obese and diabetic condition may impair MAB function due to downregulation of adiponectin and further assessment of the impact of diabetes on the functional and molecular properties of MABs will be necessary to better understand their therapeutic potential.

### PW1-expressing interstitial cells (PICs) and Twist2-dependent interstitial progenitors

In 2010, Mitchell et al. identified that cell stress mediator PW1/paternally expressed gene 3 (PW1/Peg3), a large zinc finger protein, was expressed in both satellite cells and a subset of Sca-1^pos^CD34^pos^Pax7^neg^ interstitial cells with myogenic potential [129]. The PW1-expressing interstitial cells (PICs) can efficiently contribute to skeletal muscle regeneration in vivo as well as self-renew and generate satellite cells. Interestingly, PICs require Pax7 for myogenic specification as PICs isolated from Pax7 null mice lose myogenic capacity [129]. Subsequent work by the same group demonstrated that PICs were heterogeneous and could be divided into two subgroups based on PDGFRα expression. Myogenic PICs were restricted in PDGFRα^neg^ population, whereas PDGFRα^pos^ PICs expressing white, beige, and brown fat-specific markers could give rise to adipocyte in culture [130]. Human PICs also exist as shown by Bonfanti et al. that PW1 gene was expressed at high levels in mesoangioblasts derived from human muscle biopsies, suggesting a subset of PICs corresponds to mesoangioblasts. More importantly, silencing PW1 in mesoangioblast inhibited its myogenic potential through MyoD degradation and abrogated its ability to penetrate the blood vessel wall and to engraft into damaged myofibers [131]. Altogether, these data indicate that myogenic potential of PICs is tightly regulated by myogenic transcription factors.

More recently, muscle Twist2 (Tw2) transcription factor-dependent interstitial progenitors have been characterized to be myogenic and specifically contribute to type IIb/x glycolytic fibers during adulthood and muscle regeneration [132]. Muscle fibers are highly adaptive and can switch from one kind to another under altered physiological or pathological conditions. In T2DM and/or obese patients, a significant muscle fiber-type switch from slow oxidative fibers (type I) to fast glycolytic fibers (types IIa, IIb, IIx) was observed, which was associated with reduced oxidative enzyme activity [11] and increased glycolytic metabolism [133]. It is worthwhile to study the effects of type 2 diabetes and obesity on Tw2^pos^ progenitors and to determine whether Tw2^pos^ cells contribute to fiber-type transition by de novo type IIb/x myofiber formation.

It is important to note that the above-mentioned interstitial progenitors partially overlap with each other. For example, FAPs partially overlap with PDGFRα-expressing pericytes and PICs, while pericytes might be the muscle-resident postnatal equivalent of mesoangioblasts. Recently, Yao et al. indicate that muscle-resident PDGFRβ^pos^ cells contain pericytes and PICs, and ablation of Laminin γ1 gene in these PDGFRβ expressing cells results in diminished myogenic activity and enhanced adipogenic activity [134]. These results indicate that pericytes and PICs are associated cell populations that use common mechanisms for cell fate specification. Future investigations should focus on the mechanisms that regulate the fate determination of these interstitial cells under both physiological and pathological conditions. The comparison of muscle-resident progenitors is summarized in Table 2.

## Stem cell therapies by targeting diabetic and obese muscle

Stem cell therapies have afforded promises in the treatment of chronic diseases including high-fat diet-induced obesity and T2DM [135–137]. Recent studies have shown that stem cell implantation into skeletal muscle may ameliorate diabetic symptoms. Ye and co-workers demonstrated that human skeletal myoblast (hSkM) transplantation into limb muscles of KK mouse, an animal model of T2DM, could alleviate hyperglycemia and hyperinsulinemia and improve glucose tolerance [138]. Donor hSkM survived extensively and integrated into host mouse skeletal muscles at 12 weeks after transplantation and resulted in changes of gene transcripts involved in insulin signaling pathway and mitochondrial biogenesis and function [138, 139]. It is possible that fusion of healthy hSkMs into host myofibers of T2DM recipients could enable the donor nuclei to supplement multiple genes involved in insulin-mediated glucose transport and metabolism and reverse muscle insulin resistance.

Mesenchymal stem cells (MSCs) are multipotent stem cells originated from the mesoderm and have been used for clinical trials to treat numerous diseases, including immune disorders and tissue injury [135]. Shibata et al. revealed that intramuscular injection of bone marrow-derived MSCs improved diabetic polyneuropathy in skeletal muscle [140]. Notably, MSCs were not incorporated into tissue structures of recipient animals; instead, they stimulated local production and secretion of basic fibroblast growth factor (bFGF) and vascular endothelial growth factor (VEGF), which may mediate the therapeutic effects in injected muscle. In another study, local injection of epidermal growth factor (EGF)-stimulated MSCs enhanced recovery of angiogenesis and blood flow of the ischemic hind-limb muscles of type 2 diabetic mice through modulation of the hypoxia-inducible factor (HIF), VEGF, and endothelial nitric oxide synthase (eNOS) pathways. Meanwhile, injected MSCs had been shown to differentiate into new vessels [141]. Therefore, it is likely that paracrine effects and differentiation coexist for MSCs to exert beneficial effects.

Similarly, Abrigo et al. indicated that systemic administration of bone marrow-derived MSCs improved HFD-induced skeletal muscle atrophy by inhibition of oxidative stress, myonuclear apoptosis, and ubiquitin proteasome pathway activation [142]. However, these anti-atrophic effects were neither mediated through incorporation of MSCs into myofibers nor related to obesity reversion. MSCs secrete a variety of cytokines and growth factors that could contribute to muscle repair through autocrine and paracrine activities [143]. Recent studies demonstrated that MSCs shed a large number of extracellular vesicles including microvesicles (0.1–1 mm in diameter) and exosomes (30–100 nm in diameter) into the extracellular space, which exert a novel paracrine effect through mediating cell-cell communication [144, 145]. For example, MSCs derived from placenta could enhance the myogenic differentiation of both mouse and human myoblasts isolated from mdx mice and DMD patients, respectively [146]. The therapeutic effects are mediated at least in part via exosomal secretion of miR-29 and extend to inhibition of fibrosis, decreasing creatine kinase levels and increasing utrophin expression in mdx mice [146].

In summary, MSCs are more likely acting in a paracrine fashion to modify muscle microenvironment. Notably, Sacchetti et al. argue that MSCs from different tissues differ widely in their transcriptomic signature and in vivo differentiation potential [147], which should be taken into consideration during therapeutic application. Longitudinal and further studies are also necessary to assess whether systemically delivered MSCs differentiate and incorporate into different tissues and to compare the efficacy of different routes of MSCs administration to counteract insulin resistance.

## Conclusion

Progressive loss of muscle mass, excessive intramuscular and intermuscular lipid deposition, and reduced muscle contractile activity are characteristics of degenerative muscular diseases and muscle disorders in systemic diseases like type 2 diabetes. Accumulating evidence suggest that both satellite cells and muscle-resident mesenchymal progenitors play important roles in maintaining skeletal muscle homeostasis and defects in either cell population could contribute to the pathogenesis of muscle diseases [43, 96].

Even though muscle-resident mesenchymal progenitors possess multilineage differentiation potency, they remain in an undifferentiated state under physiological condition. In injured or diseased muscle, such as in DMD or FOP muscle, these progenitors undergo lineage-specific differentiation and can adopt a fibrogenic, adipogenic, osteogenic, or chondrogenic fate (Table 2) [85, 96]. Dynamic and reciprocal interactions between satellite cells and interstitial cells or among distinct interstitial cell populations may determine the fate of interstitial progenitor cells [26, 79]. Moreover, this kind of interplay may have an impact on the direction of muscle repair towards regeneration or fibroadipogenic degeneration [80, 116].

A complete understanding of the function of diverse muscle-resident progenitors and unraveling the mechanisms underpinning their interplay and fate specification will shed light on developing new strategies to maintain muscle integrity and therapeutic interventions against obesity and diabetes.

## Abbreviations

ACVR1: Activin A receptor type I
AKT: Also known as protein kinase B
AMPK: AMP-activated protein kinase
aP2: Adipocyte protein 2
bFGF: Basic fibroblast growth factor
BMP: Bone morphogenetic protein
Bmpr1a: Bone morphogenetic protein receptor 1a
CKD: Chronic kidney disease
DMD: Duchenne muscular dystrophy
ECs: Endothelial cells
EGF: Epidermal growth factor
eIF3-f: Eukaryotic translation initiation factor 3 subunit F
eNOS: Endothelial nitric oxide synthase
FABP4: Fatty acid binding protein 4
FACS: Fluorescence activated cell sorting
FAPs: Fibroadipogenic progenitors
FOP: Fibrodysplasia ossificans progressiva
HFD: High-fat diet
Hh: Hedgehog
HIF: Hypoxia-inducible factor
HO: Heterotopic ossification
hSkM: Human skeletal myoblast
IMCL: Intramyocellular lipid
IRS-1: Insulin receptor substrate-1
LZR: Lean Zucker Rat
MABs: Mesoangioblasts
MAPK: Mitogen-activated protein kinase
MCAM: Melanoma cell adhesion molecule
MCP: Monocyte chemotactic protein
MMP14: Matrix metallopeptidase 14
MRFs: Myogenic regulatory factors
MSCs: Mesenchymal stem cells
MuRF1: Muscle ring-finger protein-1
NCAM: Neural cell adhesion molecule
NG2: Neuron-glial antigen
OZR: Obese Zucker rat
PDGFRα: Platelet-derived growth factor receptor α
Peg3: Paternally expressed gene 3
PI3K: Phosphoinositide-3-kinase
PICs: PW1-expressing interstitial cells
PIP3: Phosphatidylinositol (3,4,5)-trisphosphate
PTEN: Phosphatase and tensin homolog
SC: Satellite cell
STAT6: Signal transducer and activator of transcription 6
T2DM: Type 2 diabetes mellitus
TIMP3: Tissue inhibitor of metalloproteinases 3
Tw2: Twist2
VCAM-1: Vascular cell adhesion molecule-1
VEGF: Vascular endothelial growth factor
